# Low-dose ionizing radiation and adverse birth outcomes: a systematic review and meta-analysis

**DOI:** 10.1007/s00420-022-01911-2

**Published:** 2022-08-01

**Authors:** Brianna Frangione, Patrick Hinton, Paul J. Villeneuve

**Affiliations:** 1 Department of Neuroscience, Carleton University, 1125 Colonel By Drive, Ottawa, ON K1S 5B6 Canada; 2 Department of Public Health Sciences, Queen’s University, Kingston, ON Canada

**Keywords:** Low-dose radiation, Birth outcomes, Birth weight, Meta-analysis, Systematic review

## Abstract

**Objective:**

Ionizing radiation is a human carcinogen, and there is evidence that exposure to low-dose ionizing radiation increases the risk of adverse birth outcomes.

**Methods:**

We undertook a systematic review and meta-analysis to synthesize the research of maternal and paternal exposure to low-dose radiation on low birth weight, miscarriage, pre-term delivery, and stillbirth. Our literature search used four databases (PubMed, Environmental Index, GeoBASE, and the Cumulative Index to Nursing and Allied Health Literature). We included study populations exposed to occupational and medical sources of radiation, nuclear disasters, and those living near nuclear power plants. We considered papers published between January 1st, 1990, and June 30th, 2021. The quality of the studies was assessed, and we performed meta-analysis using random effects models to generate summary measures of association. Forest plots were created to assess the heterogeneity in these measures, and funnel plots were used to assess publication bias.

**Results:**

Overall, 26 studies were identified, and these yielded measures of association from 10, 11, and 8 studies for low birth weight, miscarriage, and stillbirth outcomes, respectively. It was not possible to perform meta-analyses for pre-term delivery due to a small number of studies. The meta-analysis summary relative risk (RR) of having a low-birth-weight infant among those ever exposed to radiation relative to those unexposed, after adjusting for publication bias, was 1.29 (95% CI 0.97–1.73). The corresponding risk estimates for miscarriage and stillbirth were 1.15 (95% CI 1.02–1.30), and 1.19 (95% CI 0.98–1.45), respectively.

**Conclusions:**

Our findings suggest that ionizing radiation increases the risk of adverse birth outcomes. Future work should strive to provide data needed to better understand the shape of the exposure–response curve.

**Supplementary Information:**

The online version contains supplementary material available at 10.1007/s00420-022-01911-2.

## Introduction

Worldwide, adverse birth outcomes account for a substantial proportion of disability and mortality. Approximately 2.6 million stillbirths occur each year globally, and an estimated 9% of all neonatal deaths are due to congenital anomalies (World Health Organization 2018). Moreover, nearly 10% of infants are born pre-term and over one million of these infants die annually as a result of premature birth (World Health Organization 2015). Although the prevalence and severity of these birth outcomes are greater in low- and middle-income countries, their prevalence has been increasing in higher-income countries (Kramer 2003; World Health Organization 2015). Adverse birth outcomes also contribute to a series of adverse health sequelae throughout the lifetime. This further highlights the need to identify etiological factors that increase the risks of these events.

The gestational period is a critical determinant of infant health and survival, and there are several indicators of infant health tied to this period, including length of gestation and birth weight (Buitendijk et al. 2003). Gestational age at birth is an indicator of organogenesis in fetal development, with normal term pregnancies lasting between 37 and 41 weeks, and pre-term birth being less than 37 weeks’ gestation (U.S. Environmental Protection Agency 2019). Low birth weight (LBW) is commonly defined as an infant being born at term with a weight less than 2500 g (< 5.5 lbs) (Kramer 2003). There are various causes of LBW in infants, including growth restriction while in utero, pre-term delivery, or both. LBW and pre-term delivery are important risk factors for short- and long-term health complications (U.S. Environmental Protection Agency 2019). These complications include infant mortality and morbidity, as well as other health effects that may present later in life, such as hypertension, diabetes, and cardiovascular disease. Low-birth weight babies have a mortality rate 25 times that of normal birth weight babies, and similarly, the mortality rate for late pre-term (34–36 weeks’ gestation) and very pre-term (< 32 weeks’ gestation) babies is three times and 75 times higher than the mortality rate for term babies, respectively (U.S. Environmental Protection Agency 2019). Another adverse birth outcome is spontaneous abortion, colloquially referred to as miscarriage (Kramer 2003). This refers to the sudden loss of pregnancy before 20 weeks’ gestation, and following 20 weeks’ gestation, sudden pregnancy loss is referred to as a stillbirth (Kramer 2003).

Exposure to ionizing radiation (IR) is ubiquitous, and from a population health perspective, most exposure occurs naturally from minerals (International Commission on Radiation Protection 2007). It has been estimated that approximately half of the general population’s non-natural exposure arises from medical procedures (International Commission on Radiation Protection 2007). Examples of these sources of radiation include those from X-ray and computed tomography (CT) scans, as well as from therapeutic radiotherapy (International Atomic Energy Agency 2020). Additionally, workers in a number of occupations such as medical professionals, miners (particularly uranium miners), nuclear power plant workers, and aircrew members, are exposed to IR (CAREX Canada 2021). Exposures are also received by those who reside near nuclear power plants, although these exposures are generally regarded as being low (Cao et al. 2022; U.S. Environmental Protection Agency 2022).

Ionizing radiation causes damage to cellular and genetic structures (e.g., DNA) in living organisms and is a recognized human carcinogen (National Research Council 2006). Much of our understanding about the adverse health effects from ionizing radiation comes from populations exposed to high doses, such as from large-scale nuclear meltdowns and from the Atomic Bomb Survivor Cohort (Davis et al. 2006; Izumi et al. 2003; Michaelis et al. 1996; Preston et al. 2008). The Biological Effects of Ionizing Radiation (BEIR) VII report of the US National Academy of Sciences defines low doses as those up to 100 mSv (National Research Council 2006). The Atomic Bomb Survivors cohort also has provided insights into the health effects of low-dose ionizing radiation given that nearly 80% of the cohort has been exposure to cumulative doses below 100 mSv (Ozasa et al. 2018). Despite a growing literature, there remain many uncertainties about the health effects of low-dose IR. An improved understanding of these risks is needed as these exposures are prevalent (Vaiserman et al. 2018).

There are several biological pathways whereby low-dose ionizing radiation may increase the risk of adverse birth outcomes. The effects of exposure can be teratogenic, carcinogenic, and mutagenic, and these risks vary based on the dosage and timing of exposure (International Atomic Energy Agency 2020). Additionally, these effects may differ with regard to maternal or paternal exposure, as there are major differences by which ionizing radiation may disrupt male and female gonads and gametes. When examining low-dose exposures, it is plausible that cell death and genetic instability occur due to the accumulation of sublethal changes, while simultaneously decreasing the efficacy of repair processes (Skrzypek et al. 2019). This may lead to genetic mutations in oocytes and spermatogonia undergoing gametogenesis, resulting in adverse embryonic and fetal outcomes (Skrzypek et al. 2019).

Herein, we sought to synthesize the published literature on low-dose radiation and the adverse birth outcomes of low birth weight, spontaneous abortions (or miscarriages), and stillbirth. Additionally, we explore whether the strength of these associations differ between maternal and paternal exposures, and between low-dose (non-therapeutic) and high dose (therapeutic) exposures. For the latter, these exposures can exceed the low-dose threshold of 100 mSv.

## Methods

### Protocol and registration

This systematic review adhered to the Preferred Reporting Items for Systematic Reviews and Meta-Analyses (PRISMA) statement (Liberati et al. 2009). Although the protocol for this systematic review was not registered, we did conduct a search of the Cochrane Library and the Prospective Register of Systematic Reviews (National Institute for Health Research 2021) for ongoing work in this area. From this registry, we did not identify any other overlapping project with this systematic review.

### Study inclusion criteria

The Population, Exposure, Comparison, Outcome (PECO) framework (Rooney et al. 2014) was followed to help plan the systematic review components. We included papers published between January 1st, 1990, and June 30th, 2021. Moreover, we also reviewed the citation lists of all identified studies to ensure that no key studies were missed, particularly those that predated our earlier inclusion date. In our view, this two-tiered approach was sufficient to identify all relevant studies to inform our synthesis.

#### Study populations

Our populations of interest included parents (either mothers or fathers) exposed to low-dose IR before conception or during pregnancy. Possible sources of exposure included: occupational, medical diagnostic, cancer treatment, or place of residence (e.g., proximity to nuclear power plant).

#### Exposure

The exposure of interest was low-dose IR to the body (any/all regions). A number of exposure sources were considered for this review. These included those exposed to nuclear disasters (e.g., Chernobyl, Hiroshima), those exposed occupationally through work in nuclear energy production, or medical radiation, as well as individuals exposed by medical/dental diagnostic techniques or radiotherapy for cancer treatment. Lastly, we considered study populations potentially exposed due to residential proximity to nuclear power plants. It is important to note that we only considered studies that measured either prior-to-conception or in-utero exposures, although radiotherapy treatment (e.g., treatment for childhood cancer) was necessarily pre-conception when assessing future risk of adverse birth outcomes in these individuals. The BEIR VII report defined low-dose radiation as exposures less than 100 mSv (National Research Council 2006). Our review adhered to this definition, however, given our interest in prevalent exposures to IR we also considered studies of therapeutic radiation that sometimes produces exposures above 100 mSv (Mehta et al. 2010).

#### Comparison

Risk estimates based on several possible comparisons were relevant for inclusion. These included: (i) comparisons between exposed and non-exposed subjects or survivors, (ii) comparisons between exposed subjects and population controls, (iii) comparisons between varying ranges of radiation exposures, (iv) comparisons between male and female exposures, (v) comparisons between targeted organs in radiotherapy, and (vi) comparisons between residential proximities and associated radiation doses.

#### Outcomes

The outcomes considered for this review were the more commonly studied and prevalent adverse pregnancy outcomes. These included: (i) low birth weight, (ii) miscarriage, (iii) pre-term delivery, and (iv) stillbirth. Additionally, subgroup analyses were performed to examine if there are differences in the associations between maternal and paternal exposures, as well as between low-dose (non-therapeutic) exposure and high dose (therapeutic) exposure, examining each outcome (excluding pre-term delivery).

#### Study design

Initially, observational cross-sectional, cohort or case–control study designs were eligible for inclusion. We included only those studies that had individual-level data for both outcome and exposure. Additionally, given our aim to conduct meta-analyses, we only included those studies that reported a measure of association (i.e., odds ratio, relative risk, incidence rate ratio), or those that supplied sufficient data for a risk measure to be calculated. Qualitative studies as well as those that only provided a graphical representation of data were excluded. Additionally, to be included in the meta-analysis, studies must have been able to control for the possible confounding influence of other risk factors.

#### Publication status and language

We restricted our search to peer-reviewed studies published in indexed journals, reports, and dissertations. We considered only studies published in English and French, as translation services for other languages were not available. We excluded non-peer-reviewed studies, reviews, other secondary sources, and grey literature (i.e., government reports, conference proceedings).

### Search strategy

The search strategy was developed by one author (BF) and reviewed for completeness by the others. One author (BF) conducted the literature search using four databases (PubMed, Environmental Index, GeoBASE, and the Cumulative Index to Nursing and Allied Health Literature). The Boolean operators (OR, AND) were used in conjunction with specific search terms, and the search strategy included the following keywords: ((Radiation) OR ("ionizing radiation" OR "radiation effects" OR "abnormalities, radiation induced" OR "low dose radiation")) AND ((pregnan*) OR ("maternal exposure" OR "birth outcome" OR "occupational exposure" OR "paternal exposure" OR "prenatal exposure")). We also examined the reference list of all included studies and relevant reviews to identify additional articles not captured with our initial search.

### Study selection

All database records were imported into EndNote X9 (The EndNote Team 2013) and de-duplicated. For the initial screening of these records, one reviewer (BF) independently screened all titles and abstracts against the inclusion criteria. A second reviewer (PH) was consulted if the first reviewer was unsure of whether to include any study. At level two screening, two reviewers (BF, PH) and independently screened full-text articles against the inclusion criteria. The senior author (PV) resolved any discrepancies. The reviewers were not blinded to the study authors when screening.

### Data extraction

We extracted data from the identified studies and entered these into a standardized Excel spreadsheet. The format of this database was developed *apriori* and reviewed by all authors. Data were extracted by two authors (BF, PH) and verified for accuracy by a third author (PV). We extracted key characteristics from each study including study design, exposure source(s), type of outcome(s), and relevant measures of association. All measures of association were interpreted as relative risks (RR) for the meta-analysis; odds ratios (OR) were assumed to be equivalent to RR as adverse birth outcomes are sufficiently rare such that the OR can be used to approximate the RR (Aschengrau and Seage 2020).

### Assessment of methodological quality

The assessment of the quality of retained studies was conducted by two independent reviewers (PH and BF) using the Joanna Briggs Institute (JBI) checklist for analytical cross-sectional studies (Moola et al. 2020). We used the cross-sectional JBI checklist regardless of study design as this checklist has been considered appropriate for assessing the quality criteria of many types of observational studies (Ma et al. 2020). The following criteria were used for rating the methodological quality of the studies: (a) good quality if the study met at least six out of the eight checklist criteria including questions five and eight; (b) moderate quality if the study met at least five of the checklist criteria; and (c) poor quality if the study met less than five of the checklist criteria.

### Statistical analyses

A meta-analysis of the measures of association was conducted using the inverse variance method and forest plots were generated (Higgins 2022). The *I*^*2*^ statistic (Higgins et al. 2003) was used to assess heterogeneity, and we considered *I*^*2*^ > 40% as moderate and *I*^*2*^ > 75% as high heterogeneity. Random-effects models were used to generate a summary measure of association across all studies. After reviewing the exposure data from included studies, it became apparent that we would not be able to standardize measures of association across studies due to different methods of exposure characterization, as well as varying cut-points. As a result, to proceed with meta-analysis, we used a dichotomous (ever versus never) classification to generate risk estimates that were used in our meta-analysis. We conducted subgroup analysis to assess whether the strength of the association was different between maternal and paternal exposure. Finally, we assessed heterogeneity in the summary measures of association between low dose (non-therapeutic) and higher (therapeutic) levels. We assessed publication bias using funnel plots and tested for statistical significance for this bias using Egger’s test (Egger et al. 1997). Where necessary, the Trim and Fill method was used to correct the summary measure of association for publication bias (Duval and Tweedie 2000). All analyses were conducted using Stata version 13 (StataCorp 2013).

## Results

### Selection and characteristics of studies

**Fig. 1:**
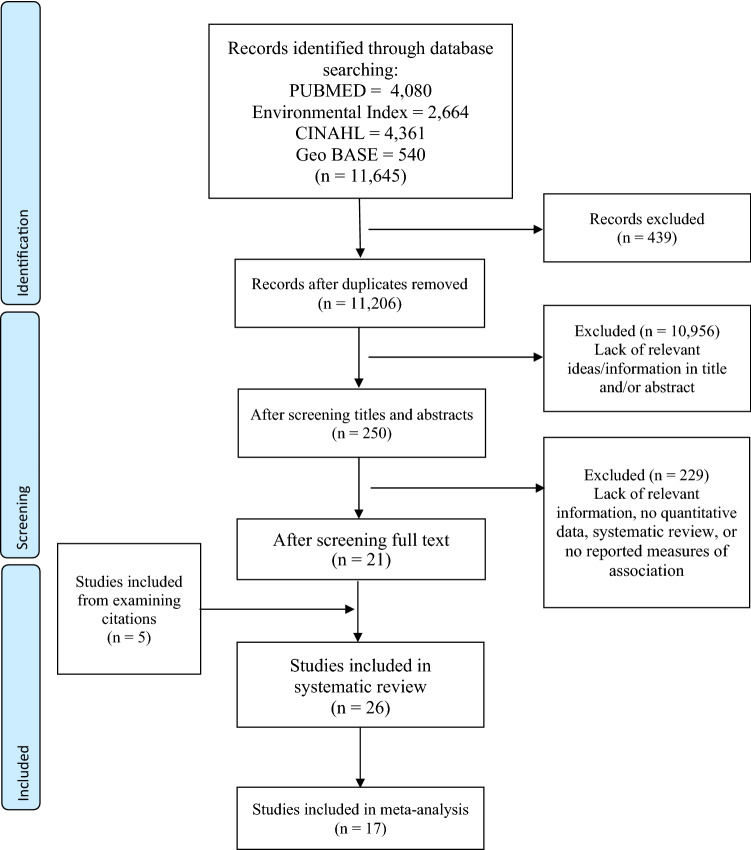
Flowchart of the studies selected for the systematic review and meta-analysis

We identified a total of 11,645 publications using our search strategy. After removing duplicates and screening for study relevance, a total of 26 studies were included in the systematic review. Of these, 17 provided sufficient information to be included in subsequent meta-analyses of the different outcomes considered (Fig. 1). The characteristics of included studies are presented in Table 1, and a description of the sources of exposure, and exposure ranges are presented in Table 2. There were 15 studies that evaluated associations between low-dose IR exposure and low birth weight, 12 that examined miscarriage, eight studies that examined stillbirth, and four studies that examined gestational age (used as a proxy for pre-term delivery). Due to the overall number of studies and the tabular data presented in the extracted studies, a meta-analysis could only be performed for low birth weight, miscarriage, and stillbirths.

**Table 1:** Characteristics of studies identified in the systematic review, and an assessment of study quality

Authors	Year	Location	Study design	Sample size	Ages	Outcome	Radiation exposure	Timing of exposure	Measure of association	Study quality assessment^*^
Andreassi et al	2020	Italy	Case–control	357	37.2 ± 5.2	LBW Miscarriage Stillbirth	Medical occupation	Paternal only exposure	Odds ratios	Moderate
Chen et al	2018	China	Retrospective cohort	192,492	25.1 ± 3.9	LBW	Medical diagnostic	Paternal only exposure	Odds ratio	Good
Chiarelli et al	2000	Canada	Cohort	668	> 18	LBW Miscarriage	Medical therapeutic	Prior to pregnancy	Odds ratios	Good
Choi et al	2013	South Korea	Age-matched cohort study	642	31.7 ± 3.8	Birth weight Gestational age	Medical diagnostic	During pregnancy	Odds ratio (excluded from meta-analysis)	Moderate
Doyle et al	2000	England	Retrospective cohort	27,261	39.8 ± 4.1	Miscarriage Stillbirth	Nuclear occupation	Prior to pregnancy	Odds ratio	Moderate
Fucic et al	2008	Croatia	Case–control cohort	462	12–51	Miscarriage	Medical occupation	Prior to pregnancy	Odds ratio	Good
Goldberg et al	1997	Canada	Retrospective cohort	2426	> 15	LBW Miscarriage Stillbirth	Medical diagnostic	Prior to pregnancy	Odds ratio	Good
Gong et al	2017	Texas, USA	Case–control	3,481,077	> 11	LBW	Residential proximity	Prior to and during pregnancy	Odds ratio (excluded from meta-analysis)	Good
Grajewski et al	2015	USA	Case–control	844	26–49	Miscarriage	Flight occupation	During pregnancy	Odds ratio	Good
Green et al	2002	Canada USA	Retrospective cohort	4029	> 15	LBW Miscarriage Stillbirth	Medical therapeutic	Prior to pregnancy	Relative Risk	Good
Green et al	2010	Canada	Case–control	1856	> 15	LBW	Medical therapeutic	Prior to pregnancy	Odds ratios derived from frequencies	Good
Guilbaud et al	2019	France	Prospective cohort	638	30.4 ± 0.7	Miscarriage Gestational age	Medical diagnostic	During pregnancy	Odds ratio	Good
Ha et al	2015	Florida, USA	Prospective cohort	423,719	26.7 ± 7.1	LBW	Residential proximity	During pregnancy	Odds ratio (excluded from meta-analysis)	Good
Hatch et al	2017	Chernobyl	Retrospective cohort	2582		Birth weight Gestational age	Nuclear disaster	During pregnancy	Parameter estimate (excluded from meta-analysis)	Good
Hujoel et al	2004	USA	Case–control	5585	12–45	LBW	Medical diagnostic	During pregnancy	Odds ratio	Good
Igumnov & Drozdovitch	2000	Chernobyl	Case–control	500		Birth weight	Nuclear disaster	During pregnancy	Slope (excluded from meta-analysis)	Moderate
Kallen et al	1998	Sweden	Cohort	19,494		LBW Stillbirth Gestational age	Medical therapeutic	Prior to pregnancy	Relative risk	Moderate
Lawson et al	2012	USA	Retrospective cohort	7482	25–42	Miscarriage	Medical occupation	During pregnancy	Odds ratio	Good
Mortazavi et al	2013	Iran	Cohort	1200	20–40	LBW	Medical diagnostic	During pregnancy	Mean + SD (excluded from meta-analysis)	Moderate
Parker et al	1999	Cumbria, UK	Cohort	251,812	Any	Stillbirth	Nuclear occupation	Paternal only exposure	Odds ratio	Moderate
Reulen et al	2009	Britain, UK	Retrospective cohort	7300	> 16	LBW Miscarriage Stillbirth	Medical therapeutic	Prior to pregnancy	Odds ratio	Good
Signorello et al	2006	Canada USA	Retrospective cohort	3376	Any	LBW	Medical therapeutic	Prior to pregnancy	Odds ratio	Good
Tsou et al	2020	Taiwan, China	Case–control	853	27.1 ± 3.8	LBW Birth weight	Residential proximity	During pregnancy	Odds ratio (excluded from meta-analysis)	Moderate
van de Loo	2019	Nether lands	Nested cohort	275	> 18	LBW Miscarriage	Medical therapeutic	Prior to pregnancy	Odds ratio (excluded from meta-analysis)	Moderate
Winther et al	2008	Denmark	Cohort	34,922	12–48	Miscarriage Stillbirth	Medical therapeutic	Prior to pregnancy	Proportion ratio	Moderate
Zhang et al	2020	Wuhan, China	Prospective cohort	8500	28.6 ± 3.7	Birth weight	Residential proximity	During pregnancy	Odds ratio (excluded from meta-analysis)	Good

*The quality of the study was assessed by the criteria described by (Moola et al. 2020)

**Table 2:** Source of radiation exposure and exposure levels of the included studies/study participants

Author	Year	Exposure	Exposure levels
Andreassi et al	2020	Occupationally exposed male workers (cardiac catheterization)	~ 1–10 mSv/year
Chen et al	2018	Paternal exposure to medical-related radiation	Not measured, anticipated to be low
Chiarelli et al	2000	Radiation exposure to treat childhood cancer	Abdominal-pelvic radiation cumulative total dose was above the 50th percentile (> 2,500 cG) was high exposure group
Choi et al	2012	Pregnant women exposed to abdominal or lumbar radio diagnostic procedures	Radiation exposures between 50 and 100 mGy (5–10 rad)
Doyle et al	2000	Nuclear industry employees	Exposure distribution not described, but those with exposures greater than 100 mSv had no excess in either sex
Fucic et al	2008	Female populations occupationally exposed to radiation	Exposures were < 10 mSv per year and no woman received a radiation dose that exceeded the international limit of 20 mSv per year or 100 mSv over 5 years
Goldberg et al	1997	Adolescent females exposed to radiation for scoliosis	Median exposure of 0.69 cGy
Gong et al	2017	Maternal residential proximity to nuclear facilities	No exposure data. Exposure categorized based on distance
Grajewski et al	2015	Occupational exposure among flight attendants	Median effective dose between 0.36 and 0.91 mSv
Green et al	2002	Pregnancy outcomes of female survivors of childhood cancer	No direct measure of exposure as contrasts made between those with radiation and other types of treatment
Green et al	2010	Radiation for treatment of Wilms tumor	Detailed exposure profile not available, however, 16% of women and 9% of men received exposures > 35 Gy
Guilbaud et al	2019	Pregnancy outcome after first trimester exposure to radiation	Median fetal dose of 3.1 mGy
Ha et al	2015	Residential proximity to power plants	No exposure data. Exposure categorized based on distance
Hatch et al	2017	Neonatal outcomes following radiation exposure in utero to fallout from Chernobyl	Cs-137 deposition levels greater than 37 kBq/m
Hujoel et al	2004	Antepartum dental radiography and infant low birth weight	Exposed group consisted of mothers with > 0.4 mGy
Igumnov & Drozdovitch	2000	Children from Belarus exposed in utero to radiation from Chernobyl accident	Mean value of thyroid doses from 131I 0.39 Gy was estimated for the prenatal exposed children
Kallen et al	1998	Outcome of reproduction in women irradiated for skin hemangioma in infancy	The mean ovarian dose was 6 cGy, and the maximum was 8.55 Gy
Lawson et al	2012	Occupational exposure among nurses	No direct measure of exposure, but rather frequency of working with X-rays was modelled
Mortazavi et al	2013	Radiation exposure in a screening program of pregnant women	No characterization of exposure provided
Parker et al	1999	Exposure among male radiation workers at Sellafield nuclear plant	The median exposure among of all live births was 0.13 mSv; the median exposure among stillbirths was 0.33 mSv
Reulen et al	2009	Radiation exposure for childhood cancer	No characterization of exposure provided
Signorello et al	2006	Radiation exposure for childhood cancer	Ovarian irradiation only among women with a dose < 100 cGy to the uterus
Tsou et al	2019	Taiwanese radiation-contaminated buildings (RCBs) natural accident	Taiwan Cumulative Dose exposure assessment system
van de Loo	2019	Radiation exposure to treat childhood cancer	Comparison of outcomes among childhood cancer survivors’ exposure to radiation to non-radiation treatments
Winther et al	2008	Radiation exposure to treat childhood cancer	Highly variable across cancer sites with exposure up to 50 Gy
Zhang et al	2020	Prenatal uranium exposure in general population	Geometric mean of U concentration of 0.03 ug/L

There were six studies that investigated occupational exposure from medical (*n* = 3) (Andreassi et al. 2020; Fucic et al. 2008; Lawson et al. 2012), nuclear (*n* = 2) (Doyle et al. 2000; Parker et al. 1999), and flight attendant (*n* = 1) (Grajewski et al. 2015) sectors. A total of 14 studies examined medical exposures for diagnostic (*n* = 6) (Chen et al. 2018; Choi et al. 2013; Goldberg et al. 1997; Guilbaud et al. 2019; Hujoel et al. 2004; Mortazavi et al. 2013) or therapeutic (*n* = 8) (Chiarelli et al. 2000; Green et al. 2010; Green et al. 2002; Källén et al. 1998; Reulen et al. 2009; Signorello et al. 2006; van de Loo et al. 2019; Winther et al. 2008) purposes. The remaining sources of exposures included those from residential proximity (*n* = 2) (Gong et al. 2017; Ha et al. 2015), nuclear disasters (*n* = 3) (Hatch et al. 2017; Igumnov and Drozdovitch 2000; Tsou et al. 2020), or environmental exposures (*n* = 1) (Zhang et al. 2020). Eleven out of the 26 studies were conducted in Europe and Central Asia, ten in North America, four in East Asia and Pacific, and one in the Middle East. By study design, there were case–control (*n* = 8) and cohort (*n* = 18) studies. Twelve studies were published between 1997 and 2010, four studies were published between 2010 and 2015, and ten studies were published since 2015.

### Association of adverse birth outcomes with IR exposure

#### Low birth weight (LBW)

**Fig. 2:**
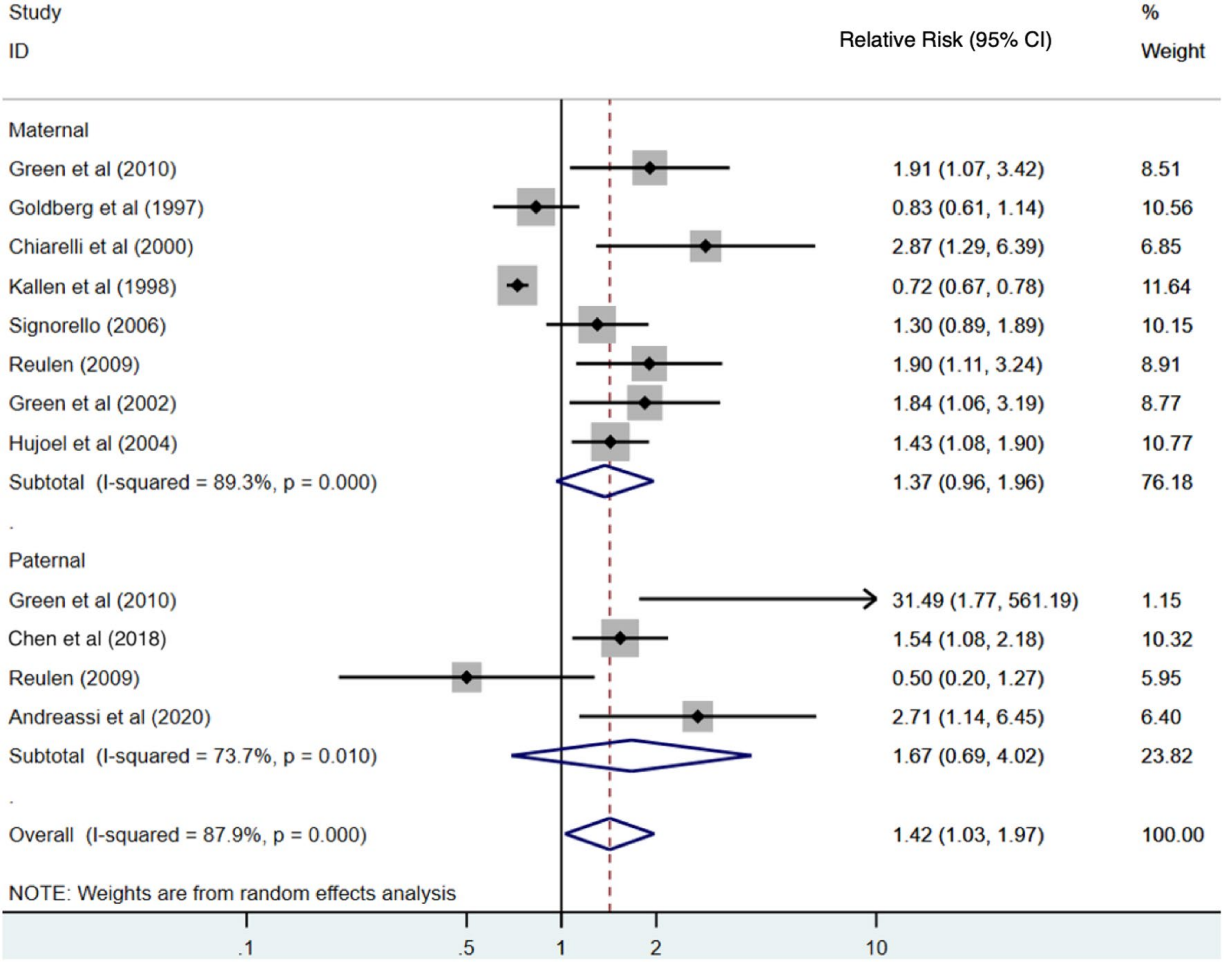
Estimates of risk of low birth weight by maternal and paternal exposure to low-dose radiation relative to those unexposed. The weights represent the contribution of each study effect estimate to the overall meta-estimate

There were ten studies that examined LBW that were included in our meta-analysis. Although the following studies were initially identified in the systematic review: Mortazavi et al (2013), van de Loo (2019), Gong et al (2017), Hatch et al. (2017), and Tsou et al. (2020); they were excluded from the LBW meta-analysis for various reasons. We excluded the (Mortazavi et al. 2013) paper because it modelled birth weight as a continuous variable and did not classify infants as being low birth weight (≤ 2500 g) or not. The (van de Loo et al. 2019) study was excluded because they did not seem to apply analyses appropriate to the matched design of the study. Three studies (Gong et al. 2017; Ha et al. 2015; Tsou et al. 2020) were excluded because individual-level exposure to radiation was not determined for the study participants. Of the included studies for the LBW meta-analysis, two studies examined occupational exposure including nuclear (*n* = 1) and medical (*n* = 1) sectors. A total of eight studies examined medical exposures for diagnostic purposes (*n* = 2) or cancer treatments (*n* = 6). The overall summary result for studies evaluating the effect of IR exposure on LBW is presented in Fig. 2. The summary measure of association from the meta-analyses was a RR of 1.42 (95% CI 1.03–1.97) (Fig. 2). A high degree of heterogeneity in the measures of association was observed across studies (*I*^*2*^ = 87.9%, *p* < 0.001).

**Fig. 3:**
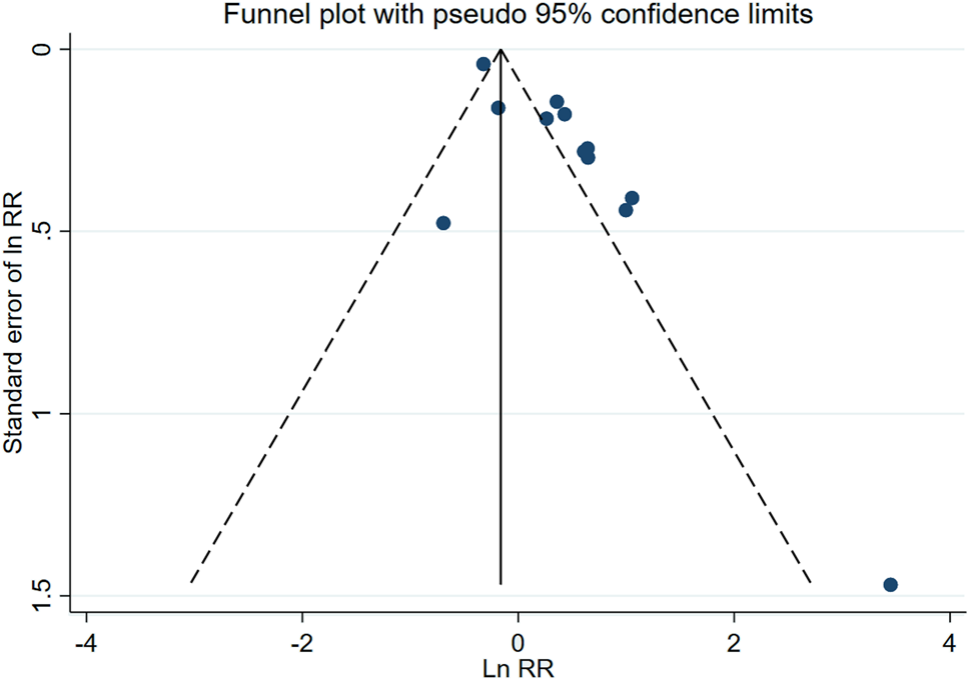
Funnel plot of risk estimates from studies that examined the association between low-dose radiation and low birth weight. Egger’s test: *z* = 3.16; Prob >|z| = 0.0016

The subgroup analysis by sex found that the summary measure of association between low-dose radiation and low-birth-weight children was higher for paternal exposures (RR = 1.67; 95% CI 0.69–4.02) than maternal exposure (RR = 1.37; 95% CI 0.93–2.02), with both having high heterogeneities of 73.7% and 89.3%, respectively. However, this difference between subgroups was not statistically significant as determined by a *z* test for the difference in relative risks (*p* = 0.69). The funnel plot of these measures of association provided some evidence of publication bias, and Egger’s test was statistically significant (*p* < 0.01) (Fig. 3). After applying the Trim and Fill method to correct for publication bias, the summary measure of association (RR) was 1.20 (95% CI 0.89–1.63) for maternal exposure and was 1.30 (95% CI 0.31–5.30) for paternal exposure. After correcting for publication bias, there was an attenuation in the measure of association (1.42 versus 1.29) and the corrected measure was no longer statistically significant. The summary measure association (both sexes combined) after correction was 1.29 (95% CI 0.97–1.73).

#### Miscarriage

**Fig. 4:**
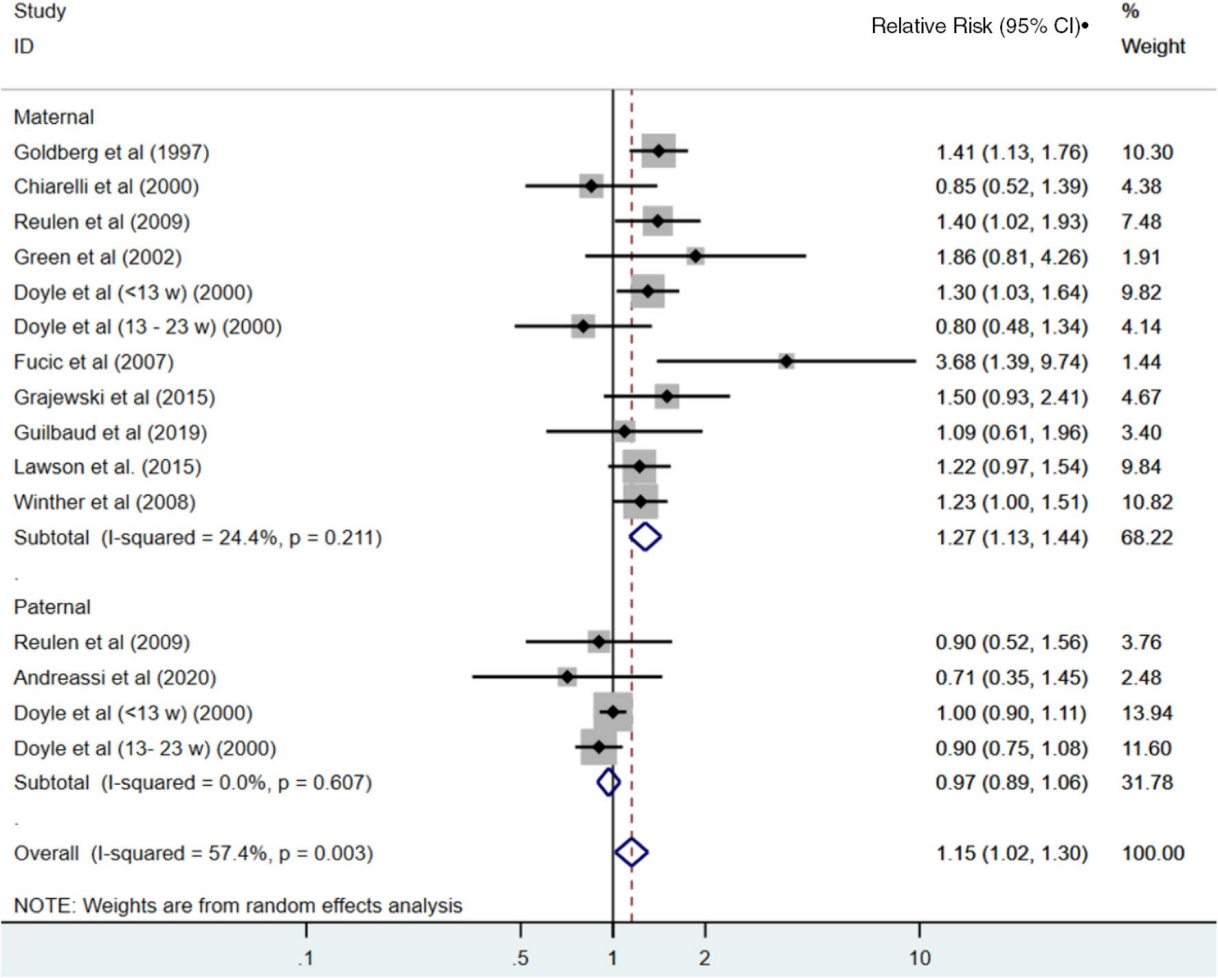
Estimates of risk of miscarriage (or spontaneous abortion) by maternal and paternal exposure to low-dose radiation relative to those unexposed. The weights represent the contribution of each study effect estimate to the overall meta-estimate

**Fig. 5:**
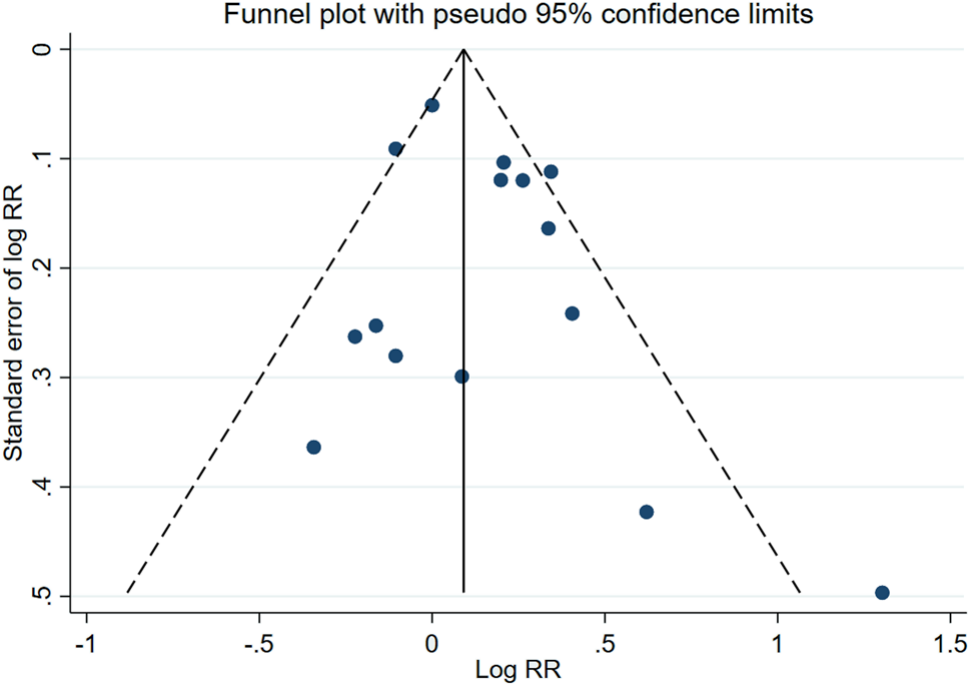
Funnel plot of risk estimates from studies that examined the association between low-dose radiation and miscarriage (or spontaneous abortion). Egger’s test: *z* = 0.77; Prob >|z| = 0.4438

Eleven studies reported associations between maternal exposure to low-dose radiation and miscarriage, while three of the studies also reported paternal exposure (Fig. 4). There was one study which reported only on paternal exposure (Andreassi et al. 2020). The (van de Loo et al. 2019) study was excluded because this paper did not apply analyses appropriate to the matched design of the study. Doyle et al. reported associations separately for miscarriages at < 13 and 13–23 weeks, and because these risks differed, we included both in the meta-analyses (Doyle et al. 2000). The summary relative risk for maternal exposure to radiation and the risk of miscarriage across these ten measures of association was 1.27 (95% CI 1.13–1.44). In contrast, there was no association observed based on the summary estimate from the studies that reported on paternal exposures (RR = 0.97; 95% CI 0.89–1.06). The overall summary relative risk, across both maternal and paternal exposures, was 1.15 (95% CI 1.02–1.30).There was no evidence of publication bias, although we note the presence of an outlier in the studies, namely the RR reported by Fucic et al. (RR = 3.68, 95% CI 1.38–8.74) (Fucic et al. 2008) (Fig. 5).

#### Stillbirth

**Fig. 6:**
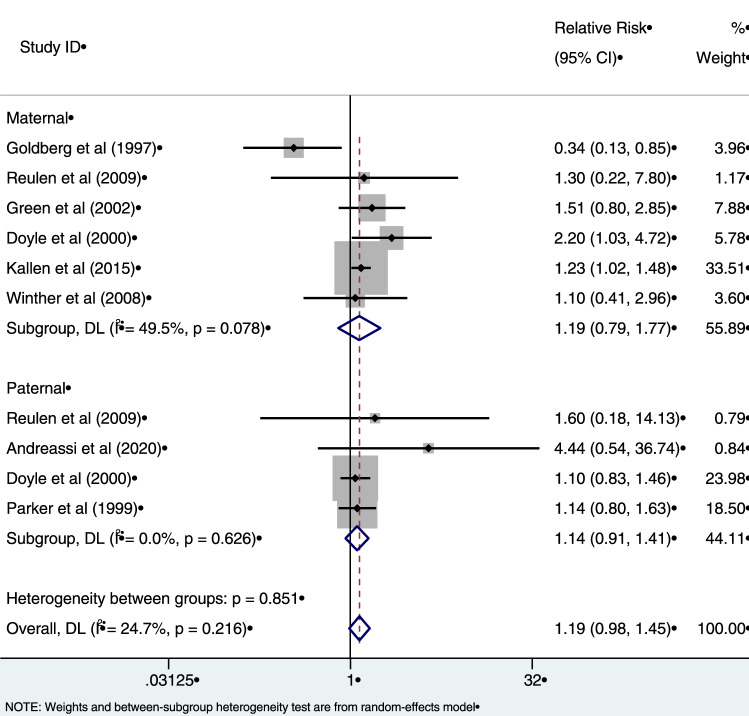
Estimates of risk of stillbirth by maternal and paternal exposure to low-dose radiation relative to those unexposed. The weights represent the contribution of each study effect estimate to the overall meta-estimate

**Fig. 7:**
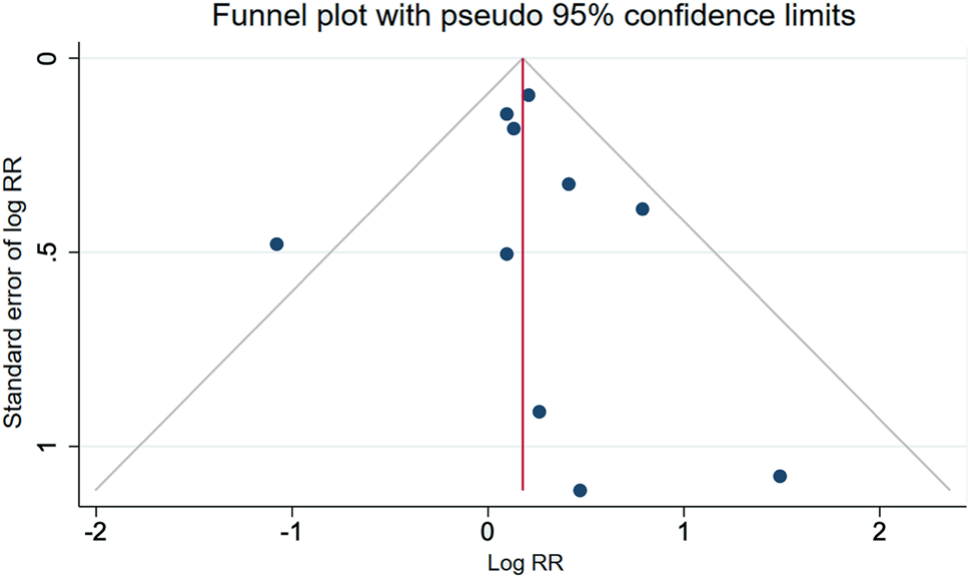
Funnel plot of risk estimates from studies that examined the association between low-dose radiation and stillbirth. Egger’s test: *z* = 0.29; Prob >|z| = 0.77

Meta-analysis was applied to eight studies that reported associations between exposure to radiation and stillbirth (Fig. 6). Six of these studies reported associations between maternal exposure to radiation and stillbirth, with two studies also reporting associations for paternal exposure. Two studies reported only paternal exposure associations (Andreassi et al. 2020; Parker et al. 1999). The summary measure of association was not statistically significant for either maternal (RR = 1.19, 95% CI 0.79–1.77), nor paternal exposure (RR = 1.14, 95% CI 0.91–1.41). There was some evidence of heterogeneity in the risk estimates for women (*I*^*2*^ = 49.5%) which was of borderline statistical significance (*p* = 0.078). The overall summary measure for both maternal and paternal exposure was 1.19 (95% CI 0.98–1.45), however, this was not statistically significant (*p* = 0.216). The funnel plot and the accompanying Egger’s test (*p* > 0.05) did not suggest evidence of publication bias (Fig. 7).

#### Gestational age

Gestational age was used as a proxy for pre-term delivery. We did not perform a meta-analysis of low-dose ionizing radiation and gestational age as there were only four studies. All of these studies characterized risks associated with maternal exposures. Choi et al. (2013) and Guilbaud et al (2019) did not report any significant differences between the exposed and control groups (Choi et al. 2013; Guilbaud et al. 2019). Hatch et al. (2017) reported a positive association between fetal irradiation dose and later delivery (Slope: 0.471 weeks/Gy, 95% CI 0.198–0.836, *p* = 0.007) (Hatch et al. 2017). The strength of the association varied with the trimester in which exposure occurred, with third trimester exposure demonstrating a statistically significant increase in gestational length (0.564 weeks/Gy, 95% CI 0.263–1.017, *p* = 0.009). Kallen et al. (1998) reported fewer than expected exposed infants born with a gestational period below 37 weeks’ (RR = 0.85, 95% CI 0.79–0.91), however, this association was not statistically significant (*p* = 0.7) (Källén et al. 1998). Together, these measures indicate that exposure decreases the risk of pre-term delivery, however, additional studies must be conducted to draw more accurate conclusions.

#### Therapeutic versus non-therapeutic exposure levels

We performed subgroup analysis to investigate differences in the association between low dose (non-therapeutic) and high dose (therapeutic) radiation exposure, for LBW (Supplementary Fig. 1), miscarriage (Supplementary Fig. 2), and stillbirth outcomes (Supplementary Fig. 3). Included in the meta-analysis for LBW, there were six studies examining irradiation from therapeutic medicine (Chiarelli et al. 2000; Green et al. 2010; Green et al. 2002; Källén et al. 1998; Reulen et al. 2009; Signorello et al. 2006), and four studies examining non-therapeutic routes (medical occupational, medical diagnostic, nuclear occupational, etc.) (Andreassi et al. 2020; Chen et al. 2018; Goldberg et al. 1997; Hujoel et al. 2004). The combined summary risk measure was 1.42 (95% CI 1.03–1.98), and statistically significant. However, there was high heterogeneity observed (*I*^*2*^ = 88.3%). The meta-analysis for miscarriage included four studies examining therapeutic exposure (Chiarelli et al. 2000; Green et al. 2002; Reulen et al. 2009; Winther et al. 2008), and seven studies examining non-therapeutic exposure (Andreassi et al. 2020; Doyle et al. 2000; Fucic et al. 2008; Goldberg et al. 1997; Grajewski et al. 2015; Guilbaud et al. 2019; Lawson et al. 2012). The combined summary risk measure of miscarriage was 1.15 (95% CI 1.02–1.30), and statistically significant. Moderate heterogeneity was observed (*I*^*2*^ = 56.7%). Furthermore, the meta-analysis for stillbirth included four studies examining therapeutic irradiation exposure (Green et al. 2002; Källén et al. 1998; Reulen et al. 2009; Winther et al. 2008), and four studies examining non-therapeutic exposure (Andreassi et al. 2020; Doyle et al. 2000; Goldberg et al. 1997; Parker et al. 1999). The combined summary risk measure of stillbirth was 1.19 (95% CI 0.98–1.45), and borderline statistically significant. It should also be noted that the outcomes included both paternal and maternal exposures, as there were a limited number of studies, and the risk measures could not be further stratified.

## Discussion

Summary risks of adverse birth outcomes following exposure to ionizing radiation were derived using data from 17 epidemiological studies, and when both maternal and paternal exposures were considered 26 measures were in our meta-analysis. Overall, we found that these exposures to ionizing radiation increased the risks of LBW babies, although this association is narrowly non-significant. Additionally, a positive association was found for miscarriages, but this association was only observed from maternal exposures, and not paternal exposures. A positive association was also found for stillbirths, but this was not statistically significant. The strength of the associations differed slightly between maternal and paternal exposures, however, the small number of studies examining paternal exposures makes any comparisons difficult.

While our findings suggest that low level ionizing radiation increases the risk of several adverse birth outcomes, it should be noted that there were substantial differences in the range of exposure concentrations across studies. As a result, it is not straightforward to compare measures of association across studies especially given we relied on ‘ever’ versus ‘never’ exposure groups. Additional research that provides more detailed data across refined exposure categories is needed. That said, it should be noted that our measures of association were not substantially different when we compared summary risk estimates between the higher therapeutic and the lower non-therapeutic sources of exposures.

In addition to varying exposure concentrations, the studies also differed with regard to the timing of the exposure. For example, the identified studies included those who underwent radiotherapy for the treatment of childhood cancer, then subsequently became pregnant in their adult years, as well as those with more recent exposures from occupation, or during pregnancy. Andreassi et al (2020) for example, suggested that the risk of subsequent adverse birth outcomes may be higher for exposures received in the 10 weeks before conception, relative to exposures received before this time (Andreassi et al. 2020). This may be due to the fact that spermatogonia lose the protective effects of Sertoli cells during maturation to spermatozoa cells and as they travel the female reproductive tract (Aitken and De Iuliis 2009).

For low birth weight, we found slightly stronger risks of adverse birth outcomes for paternal rather than maternal exposure. However, these sex-differences were not statistically significant. There are several factors to consider when examining sex differences in radiosensitivity including the stage of gametogenesis when exposure occurred, as well as the various pathways in DNA repair mechanisms between the male and female germ cells (Eichenlaub-Ritter et al. 2007). Additionally, among both men and women, radiosensitivity depends on the type of radiation, irradiation dose, time of exposure, type of cell that was irradiated, and the phase of cell division during exposure (Wdowiak et al. 2019). It has been observed that radiation-induced biological effects do not result exclusively from cells or DNA being exposed to IR, but also the cells that have not been directly irradiated, a phenomenon known as non-targeted effects (Mavragani et al. 2016). Non-targeted effects may occur from low-dose exposure (≤ 1 Gy) and thus, creates cause for concern as these low doses are equivalent to environmental, chronic IR exposure (Mavragani et al. 2016). Based on animal studies, changes in fertility parameters (e.g., reduction in number of oocytes or spermatogonia, ovarian failure, uterine growth restriction), are not associated with the irradiated species, but rather with the exposure dose and duration (Skrzypek et al. 2019). The effects of IR exposure exceed gonadotoxic changes and may also contribute to altered uterine vascularization, decreased uterine volume and elasticity, and endometrial insufficiency (Skrzypek et al. 2019). These physiological changes contribute to pregnancy complications including pre-term delivery, low birth weight, as well as uterine rupture and stillbirth (Skrzypek et al. 2019). Furthermore, radiosensitivity of the ovaries is highly dependent on the age of women exposed to IR, with younger females experiencing greater damage via irradiation (Skrzypek et al. 2019). There are three major mechanisms occurring in DNA damage which are chromatin remodelling, oxidative stress, and apoptosis (or cell-directed senescence), and these mechanisms do not occur in a mutually exclusive manner, and the aetiology of adverse birth outcomes relating to IR exposure is highly multifactorial (Aitken and De Iuliis 2009).

Length of gestation is an important factor to consider when examining low birth weight. Due to the nature of the morbidities associated with immature development and growth, LBW babies often overlap with pre-term births. Preterm low birth weight includes infants born with a weight between 1501 and 2499 g and < 37 weeks’ gestation, whereas term low birth weight includes infants born with a weight between 1501 and 2499 g and ≥ 37 weeks’ gestation. In terms of outcome validity, using term birth weight as a measure is preferred, however, due to the limited number of studies adjusting for this difference (Chiarelli et al. 2000; Hujoel et al. 2004; Mortazavi et al. 2013; Reulen et al. 2009), our summary measure was calculated using birth weight regardless of pregnancy term. Ideally, future studies would provide risks of low birthweight among those born at full term.

Regarding exposure following nuclear disasters, a study by Scherb et al. found an increase in LBW infants in Japan in 2012 following the Hiroshima and Nagasaki accident (Scherb and Hayashi 2020). We excluded this study because the analysis was unable to control for individual-level risk factors for low birth weight, as well as lack of information on individual-level exposure. Additionally, a reanalysis using an updated dosimetry method of the results from a large cohort of atomic bomb survivors indicates an increased incidence of stillbirths following radiation exposure (Otake et al. 1990). However, the major focus of this study was examining different dosimetry methods and did not provide relevant measures of association to be included in our meta-analysis.

There is public interest in the health effects that accompany living near nuclear power plants. We identified only two studies that reported on risks of adverse birth outcomes based on residential proximity to these facilities (Gong et al. 2017; Ha et al. 2015). The Ha et al., study provides some evidence for an increased risk of low birth weight but not pre-term delivery among those who lived within 20 km of a nuclear power plant compared to those who lived further away (Ha et al. 2015). The finding for low birth weight (RR = 1.37; 95% CI 0.81–2.31) was not statistically significant. The study was unable to account for daily activities of the mothers as well as residential mobility during pregnancy. The Gong et al., study reported no statistically significant association between residential proximity and risk of LBW infants, and likewise to the Ha et al., study, this study was excluded from the meta-analysis due to the lack of individual-level data. Previous work by Hystad et al. in 2014 suggests a substantial proportion of women move during pregnancy, and thus this may introduce exposure measurement error (Hystad et al. 2014). A study conducted by Mangones and colleagues also found no association between distance to nuclear power plants and low birth weight (Mangones et al. 2013), however, this study was excluded due to its ecological design. In conclusion, there is a need for further investigation into residential exposure to radiation and the potential impacts on adverse birth outcomes. Additionally, these studies must have adequate control settings and be able to provide individual-level exposure data.

Publication bias is an important consideration when conducting meta-analysis. This bias arises because studies with positive findings are more likely to be published than those with null findings. We found some evidence of publication bias for studies of low birth weight, and after adjusting for this bias the summary measure of risk was still elevated but did not attain statistical significance. We found no evidence of publication bias for the other outcomes considered (excluding low versus high dose subgroup analysis); however, we recognize we have limited power to assess this bias given the small number of studies.

## Conclusion

In conclusion, our systematic review and summary measures adds to the growing literature that suggests exposure to low-dose ionizing radiation may increase the risk of some adverse birth outcomes. Overall, the strength of the associations were relatively modest and often statistical significance was not achieved. We observed substantial heterogeneity in the published risk estimates across studies. This may be due to a number of factors including different sources of exposure, varying exposure concentrations, different study designs, and the ability to adjust for other confounding factors. Future research is needed to provide data that better allows for the characterization of the exposure–response curve. We recognize that our findings are limited by the reliance of a dichotomous measure of exposure. An improved understanding of the etiological role of low-dose ionizing radiation may help to inform future maternal and fetal public health decisions.

## Supplementary Information

Below is the link to the electronic supplementary material.

Supplementary file1 (DOCX 519 KB)
