# Supplementary material for: Low-dose ionizing radiation and adverse birth outcomes: a systematic review and meta-analysis
Source: Int Arch Occup Environ Health. 2022 Aug 1;96(1):77–92. doi: 10.1007/s00420-022-01911-2 (PMC9823032; doi:10.1007/s00420-022-01911-2)
Supplement: Supplementary file 1 — Supplementary file1 (DOCX 519 KB) [file 420_2022_1911_MOESM1_ESM.docx]

**
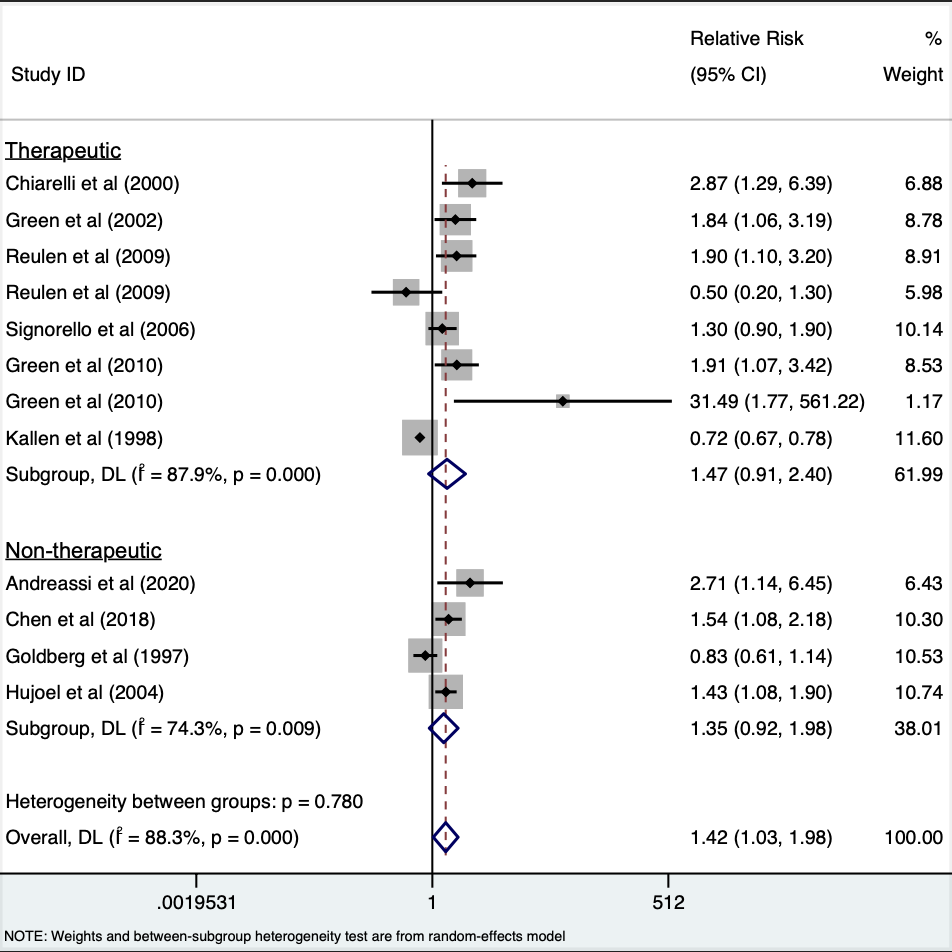
Supplementary Fig. 1** Estimates of risk of LBW by low dose (non-therapeutic) and high dose (therapeutic) exposure to ionizing radiation relative to those unexposed. The weights represent the contribution of each study effect estimate to the overall meta-estimate.

**
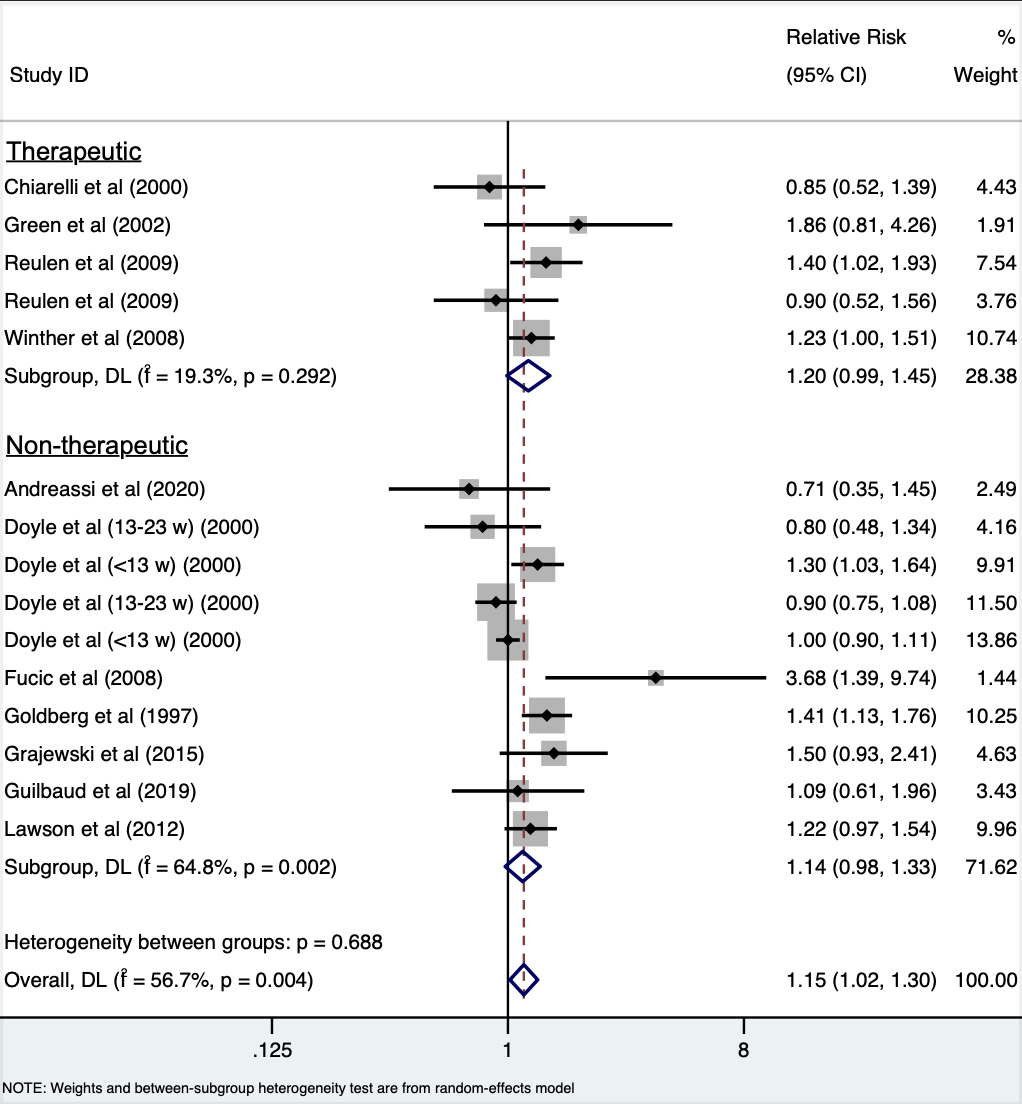
**

**Supplementary Fig. 2** Estimates of risk of miscarriage by low dose (non-therapeutic) and high dose (therapeutic) exposure to ionizing radiation relative to those unexposed. The weights represent the contribution of each study effect estimate to the overall meta-estimate.

**
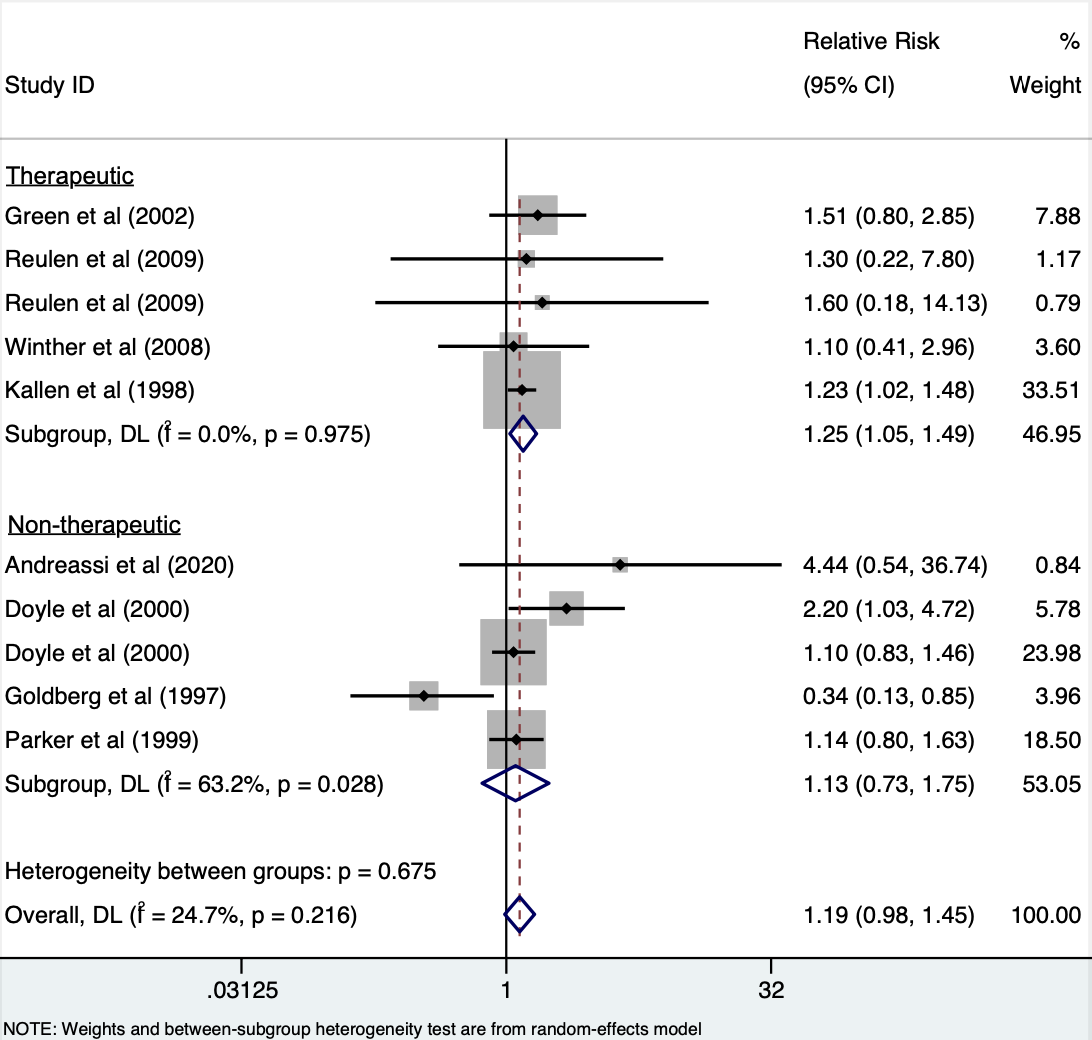
**

**Supplementary Fig. 3** Estimates of risk of stillbirth by low dose (non-therapeutic) and high dose (therapeutic) exposure to ionizing radiation relative to those unexposed. The weights represent the contribution of each study effect estimate to the overall meta-estimate.
